# *MTHFR* C677T polymorphism interaction with heavy alcohol consumption increases head and neck carcinoma risk

**DOI:** 10.1038/srep10671

**Published:** 2015-06-02

**Authors:** Xianlu Zhuo, Jue Song, Dairong Li, Yongzhong Wu, Qi Zhou

**Affiliations:** 1 Department of Radiation Oncology, Chongqing Cancer Institute, Chongqing, China; 2 Physical Examination Center, Affiliated Hospital of Guiyang Medical College, Guizhou, China; 3 Department of Medical Oncology, Chongqing Cancer Institute, Chongqing, China; 4 Department of Gynecologic Oncology, Chongqing Cancer Institute, Chongqing, China

## Abstract

MTHFR C677T polymorphism has been indicated to be a risk factor for cancers, but its association with head and neck cancer (HNC) risk remains inconclusive. In the present study, we aimed to get a more precise estimation by performing a quantitative meta-analysis. Published papers up to Jun 2014 was searched and screened. Necessary information was rigorously extracted for data pooling and analyzing, and then, subgroup analyses on ethnicity, source of controls, sample size, tumor type, smoking and drinking status were also carried out. As a result, twenty-three case-control studies including 14298 subjects were included. The overall data failed to reveal a significant association between MTHFR C677T polymorphism and HNC risk (homozygote comparison model: OR = 1.16; 95%CI = 0.93-1.45; dominant model: OR = 1.05; 95%CI = 0.90-1.21; recessive model: OR = 1.14; 95%CI = 0.93-1.38). However, in the subgroup analysis about drinking status, increase risk was shown in the heavy drinking subgroup (TT vs CC: OR = 3.11; 95%CI = 1.52-3.02). In conclusion, the results of the present study suggest that Homozygous TT alleles of MTHFR C677T polymorphism might be a risk factor for HNC among individuals who have a heavy drinking history. Further studies are needed to get a more definitive conclusion.

Head and neck carcinoma (HNC), the sixth most frequent kind of cancer worldwide, is a group of biologically similar cancers that originate from head and neck regions such as oral cavity, pharyngeal cavity, and larynx^1^. Previous reports showed that life-style factors such as smoking, drinking, betel quid chewing, papilloma virus infection, and exposure to toxic substances are possible etiological risk factors for HNC^2^,^3^. Nevertheless, though many individuals are exposed to these external factors, HNC develops only in a small proportion of the exposed people, indicating that intrinsic factors such as genetic polymorphism might play critical roles in its carcinogenic mechanisms.

Generally, folate plays a fundamental role of providing methyl groups for *de novo* deoxynucleoside synthesis and for intracellular methylation reactions in humans^4^. Low folate levels may result in uracil misincorporation during DNA synthesis, impaired DNA repair and chromosomal damage^5^. Several key enzymes were involved in the folate metabolism and their functions may have an effect on folate levels and DNA methylation. Methylenetetrahydrofolate reductase (*MTHFR*), a key enzyme for intracellular folate hemeostasis and metabolism, catalyses the irreversible conversion of 5,10-methylenetetrahydrofolate to 5-methyltetrahydrofolate that is the primary circulating form of folate, providing methyl groups for the methylation of homocysteine to methionine^6^,^7^. A common variation of *MTHFR* (rs1801133), C677T in exon 4 (Ala222Val), may be implicated in tumorigenesis with alteration of *MTHFR* enzyme activity^8^. The homozygous genotype 677TT has been indicated to have only 30% of the *MTHFR* enzyme activity of the 677CC wild-type genotype^9^. Therefore, alteration of the enzyme activity resulted from the polymorphism has been thought to be associated with cancer progression and development^10^.

Published data on the association of *MTHFR* polymorphism with HNC have generated inconclusive results. Clarifying this association may help us better understand the possible risk of HNC and therefore contribute to its prevention. Previously, we assessed the relationship between *MTHFR* polymorphism and oral cancer risk^11^. Recently, several original studies have been reported. Thus, in the present study, we performed an updated meta-analysis including the recent investigations that were conducted on head and neck cancers.

## Materials and methods

### Literature search strategy

The meta-analysis was presented according the PRISMA-MOOSE statement. We carried out a search in the internet covering well-known biomedical databases such as Medline, EMBASE, and CNKI without a language limitation. Papers published up to Jun 1, 2014 were included. The literature selection was performed from Jun 1, 2014 to Jun 30, 2014. The following keywords were used for searching: *methylenetetrahydrofolate reductase, MTHFR, head and neck, oral, pharynx, larynx, thyroid, mouth, neoplasm, tumor, cancer, variation* and *polymorphism*. All searched studies were retrieved and the bibliographies were checked for other relevant publications. Review articles and bibliographies of other relevant studies identified were hand searched to find additional eligible studies.

### Inclusion criteria

The following criteria were used for the literature selection: First, studies should concern the association of *MTHFR* C677T polymorphism with HNC risk; second, studies should be case-control or cohort designed; third, papers must offer the size of the sample, odds ratios (ORs) and their 95% confidence intervals (CIs), the genetic distribution or the information that can help infer the results. After deliberate searching, we reviewed all papers in accordance with the criteria defined above for further analysis.

### Data extraction

Information was carefully extracted from all eligible papers by two of the authors (XZ and JS) independently according to the inclusion criteria mentioned above. If their results were conflicting, an agreement was reached following a discussion. If a consensus could not be reached, another author (DL) was consulted to resolve the dispute and then a final decision was made by the majority of the votes. The extracted data were entered into a database.

### Statistical analysis

The odds ratio (OR) of *MTHFR* C677T polymorphisms and HNC risk was estimated for each study. The pooled ORs were performed for a homozygote comparison model (TT versus CC), a dominant model (TT + CT versus CC) and a recessive model (TT versus CT + CC), respectively. For detection of any possible sample size biases, the OR and its 95% confidence interval (CI) to each study was plotted against the number of participants respectively. For assessment of the heterogeneity between studies, two indexes were calculated. One was *I*^*2*^ metric, with *I*^*2*^ = 0-25% indicating no heterogeneity, *I*^*2*^ = 25-50% indicating moderate heterogeneity, and *I*^*2*^ > 50% indicating large heterogeneity^12^. The other index was a Chi-square based Q statistic test. If the result of the Q-test was *P* > 0.1 (indicating no heterogeneity), ORs were pooled according to the fixed-effect model (Mantel-Haenszel), Otherwise, the random-effect model (DerSimonian and laird) was used. The significance of the pooled ORs was determined by Z-test. The Hardy-Weinberg equilibrium (HWE) was assessed via Fisher’s exact test. Publication bias was assessed by visual inspection of funnel plots^13^, in which the standard error of log (OR) of each study was plotted against its log (OR). The symmetry of the funnel plot was further evaluated by Egger’s linear regression test^14^. An asymmetric plot indicates a possible publication bias. Statistical analysis was conducted by using the STATA 11.0 software (Stata Corporation, Texas).

## Results

### Study characteristics

**Figure 1:**
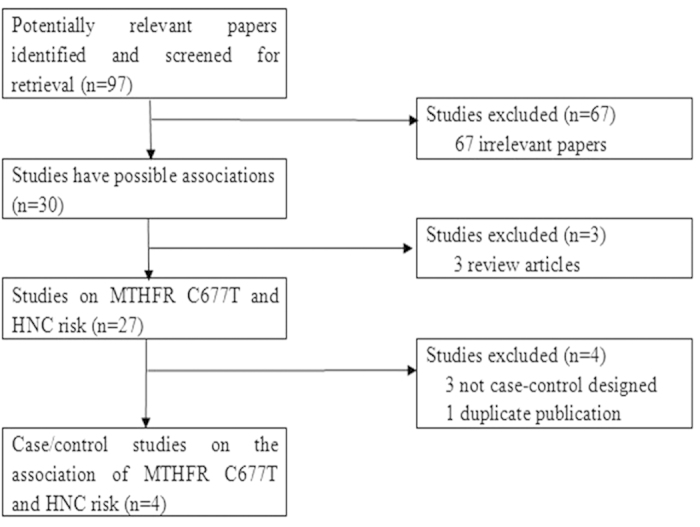
A flow chart of the literature screening.

Publications relevant to the key words were retrieved and screened originally. A total of ninety-seven studies were searched and screened for retrieval, of which sixty-seven irrelevant studies were excluded. Then, three review articles^15^,^16^,^17^ were excluded. Next, three studies that were not case-control designed^18^,^19^,^20^ (also not cohort designed) were discarded. Thus, a total of twenty-four papers were included for data extraction. Nevertheless, one duplicate publication^21^ was further excluded. Lastly, twenty-three case-control studies^22^,^23^,^24^,^25^,^26^,^27^,^28^,^29^,^30^,^31^,^32^,^33^,^34^,^35^,^36^,^37^,^38^,^39^,^40^,^41^,^42^,^43^,^44^ were selected (Fig. 1).

All the included studies were written in English except for one study in Chinese^30^. We established a database according to the extracted information from each article. The relevant information was listed in Table 1. According to the lists, the first author and the number and characteristics of cases and controls for each study as well as other necessary information were presented. As shown in this table, the selected studies involved cancers originated from oral cavity, pharynx, larynx and thyroid.

In the included studies, there were eight groups of Caucasians^23^,^27^,^29^,^33^,^38^,^41^,^42^,^43^, eight of Asians^22^,^30^,^32^,^35^,^37^,^39^,^40^,^44^ and seven of mixed populations^24^,^25^,^26^,^28^,^31^,^34^,^36^. Information about smoking could be extracted from five studies^22^,^25^,^30^,^39^,^40^ and data regarding drinking status were also available from five studies^23^,^25^,^37^,^38^,^39^. The distributions of *MTHFR* genotype of the included studies were also presented (Table 2). Of note, the data about CT and TT in the study by Hsiung *et al.*^26^ were combined as TT + CT, and therefore, the relevant data were only included in the dominant model for the whole evaluation. The genetic distributions of the control groups were consistent with HWE except for those in four studies^24^,^33^,^41^,^42^.

### Meta-analysis results

We evaluated the heterogeneity for the homozygote comparison model (TT versus CC), dominant model (TT + CT versus CC) and recessive model (TT versus CT + CC), respectively. As shown in Table 3, the heterogeneity for the overall data was significant in the three models, respectively, because the P value was less than 0.1 for Q-tests and I-squared values indicated more than moderate heterogeneity. Therefore, the random-effect models were used in the present meta-analysis.

**Figure 2:**
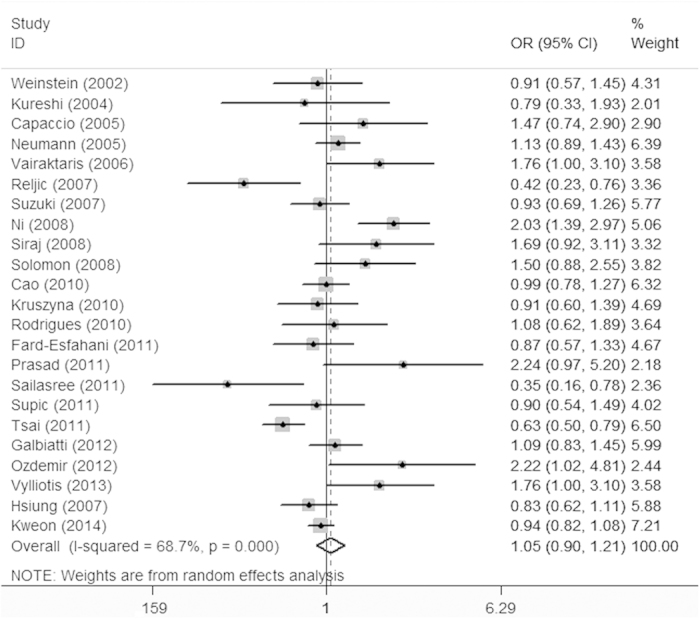
Meta-analysis for the association of HNC risk with *MTHFR* C677T polymorphism (TT + CT versus CC; overall data).

Table 3 lists the main results of the meta-analysis. The overall data in the homozygote comparison model (OR = 1.16; 95%CI = 0.93-1.45), the dominant (OR = 1.05; 95%CI = 0.90-1.21) and the recessive models (OR = 1.14; 95%CI = 0.93-1.38) failed to reveal a marked association between *MTHFR* C677T polymorphism with HNC risk (Fig. 2).

Similar results were observed in the subgroups regarding ethnicity, source of controls, sample size, tumor type and smoking status. Nevertheless, in the subgroup analysis concerning drinking status, elevated risk was shown in the heavy drinking subgroup under the homozygote comparison model (OR = 3.11; 95%CI = 1.52-3.02), indicating that TT carriers who have a heavy drinking history might have an increased HNC risk.

### Sensitivity analysis and Bias diagnostics

To test the stability of the overall results, we first excluded the studies whose genetic distributions in controls markedly deviated from HWE, given that the deviation might result in any bias^45^. The significance of the overall data in the three models was also not statistically altered. Then, we also conducted one-way sensitivity analysis^46^ by deleting a single study each time. The statistical significance of the results was not also altered (data not shown), confirming the stability and credibility of the results.

**Figure 3:**
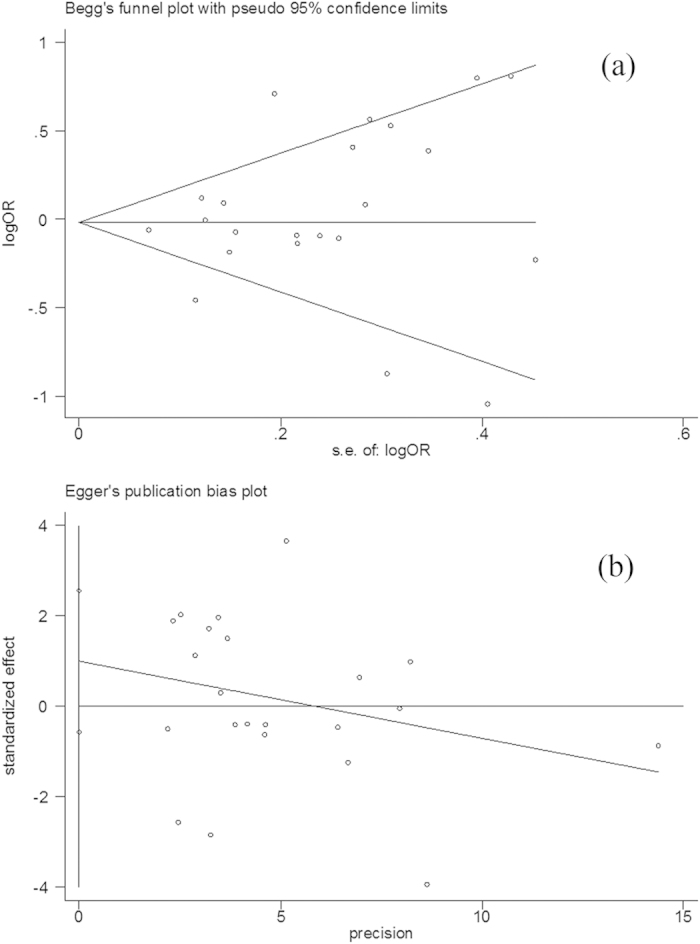
Publication bias test for the overall data (TT + CT versus CC). (a) Funnel plot; (b) Egger’s linear regression test.

Funnel plots were created for assessment of possible publication bias. Then, Egger’s linear regression tests were used to assess the symmetry of the plots. As a result, the data suggest that the funnel plots were symmetrical for the three models (homozygote comparison model: t = 0.13, P > 0.05; dominant model: t = −1.29, P > 0.05; recessive model: t = 0.87, P > 0.05), suggesting that the publication bias may have little influence on the results (Fig. 3).

## Discussion

The effects of *MTHFR* C677T polymorphism on cancer risk were controversial. Recent published meta-analyses showed that 677 C > T variation might contribute to the development of breast and esophageal cancer^47^,^48^; however, a decreased cancer risk was observed in colorectal cancer^49^. Thus, *MTHFR* C677T polymorphism may play various roles in different carcinomas. In the present study, though the overall results failed to suggest a relationship between *MTHFR* C677T variation and HNC risk, the subgroup analysis indicated that homozygous 677TT might be associated with increased susceptibility to HNC in individuals who have a heavy drinking history.

Considering the possible effects of the confounding factors on the overall data, we tried to carry out subgroup analyses. In subgroup analysis according to ethnicity, no significant association between *MTHFR* 677T allele with HNC risk was observed among the three subgroups. The results were in line with the overall data, indicating that the effects of discrepancies among different ethnicities on the results were not evident.

When the data were stratified by source of controls and sample size, respectively, the results were not significantly different from the whole results, indicating that these two factors have little influence on the results. Likewise, in the subgroup stratified by tumor type, the results were in accordance with the overall data, implying that the dissimilarity of tumor types rarely affect the pooled results.

Whether chronic smoking interacts with folate status in the pathophysiologic process of disorders remains controversial^50^. Evidence suggests that low serum folic acid concentrations were commonly detected in smokers^51^. Nevertheless, cytological damage, an early event of carcinogenesis, is evident in the mouths of smokers, but it dose not correlate with folate status^52^. In the present meta-analysis, data about smoking status could be extracted from five included studies. The results showed that elevated risk was observed in neither the never-smoking subgroup nor the ever-smoking subgroup. The data failed to reveal an obvious interaction of smoking and *MTHFR* variation for HNC risk. However, when the data were divided by drinking status, elevated risk was shown in the heavy drinking subgroup under the homozygote comparison model. The data were in line with our previous meta-analysis about oral cancer^11^. The underlying mechanisms were unclear. *MTHFR* 677C → T polymorphism might play different roles for cancer risk according to the folate levels. It might reduce cancer risk when folate status is normal^53^ while lead to impaired stability and reduced activity of the enzyme under low folate conditions^54^. Notably, exposure to alcohol consumption might result in low folate status because chronic alcohol exposure may interfere with folate absorption by suppressing the folate carrier expression, thus decreasing the expression of folate transport proteins and reducing the hepatic uptake and renal conservation of circulating folate^55^,^56^. Also, alcohol might act as a folate antagonist that is responsible for abnormalities in folate-mediated one-carbon metabolism^57^. Moreover, folate depletion in mitochondria caused by chronic alcohol exposure might lead to abnormal DNA synthesis and DNA methylation that has been thought to be involved in the carcinogenesis process^58^,^59^. This might explain the interactions of alcohol with 677TT genotype in the genesis of HNC. However, only five of the selected studies concerned this issue. In the meantime, the data should be interpreted with care because of the limited sample sizes.

Evident heterogeneity was shown in every model for the overall data, and thus, random-effect models were used to pool the data. Nevertheless, significant heterogeneities were removed in some subgroups while observed in other subgroups, indicating that the heterogeneities may be multifactorial. Besides the confounding factors considered in the subgroup analysis, other factors such as age, gender and life-style factors might contribute to the heterogeneities.

Several limitations might be involved in this meta-analysis. First, only publications written in English and Chinese were searched and selected. It is possible that articles written in other languages that might meet the inclusion criteria were missed. Thus, inevitable publication biases might exist though the funnel plots were tested to be symmetrical. Second, subgroup analyses regarding age, gender and other risk factors such as virus infection status and betel quid chewing have not been conducted because the data in the primary literature were insufficient. Third, hospital-based controls were used in some of the included studies, leading to non-differential misclassification bias. Moreover, the controls in some studies were not strictly matched to the cases. Therefore, any selection bias might have an influence on the overall results and future investigations with large sample sizes are required.

Despite the limitations, though the overall data of the present meta-analysis did not reveal an association of *MTHFR* C677T polymorphism with HNC risk, the subgroup analysis indicated that *MTHFR* 677TT alleles might increase HNC risk in individuals who have a heavy drinking history.

## Additional Information

**How to cite this article**: Zhuo, X. *et al.* *MTHFR* C677T polymorphism interaction with heavy alcohol consumption increases head and neck carcinoma risk. *Sci. Rep.* **5**, 10671; doi: 10.1038/srep10671 (2015).

## Figures and Tables

**Table 1:** Characteristics of studies included in the meta-analysis.

**First Author**	**Publication Year**	**Number of Cases (male/female)**	**Number of Controls (male/female)**	**Type of controls**	**Mean age, year Cases/Controls**	**Racial decent**	**Types**	**Country**	**Ref No.**
Weinstein	2002	519 (NA/NA)	629 (NA/NA)	Healthy controls (Age-, gender- matched; population-based)	63.2/61.0	Caucasian	Oral cavity	USA	43
Kureshi	2004	50 (33/17)	54 (30/24)	Healthy controls (Age-, gender- matched; population-based)	51.3/50.2	Mixed	Combined	Pakistan	28
Capaccio	2005	65 (57/8)	100 (88/12)	Healthy controls (Age-, gender-, drinking-, smoking status- matched; population-based)	61.3/58.8	Caucasian	Combined	Italy	23
Neumann	2005	537 (411/126)	545 (401/144)	Non-cancerous controls (Age-,sex-, smoking status-matched; hospital-based)	NA/NA	Caucasian	Combined	USA	29
Vairaktaris	2006	110 (94/16)	120 (102/18)	Healthy controls (Age-, sex, ethnicity-matched; population-based)	52.1/51.5	Caucasian	Oral cavity	GreekGerman	41
Hsiung	2007	278 (193/85)	526 (377/149)	Healthy controls (Age-, gender, town of residence-matched; population-based)	60.1/61.0	Mixed	Combined	USA	26
Reljic	2007	81 (NA/NA)	102 (37/65)	Healthy controls (Age-matched; population-based)	NA/NA	Caucasian	Combined	Croatia	33
Suzuki	2007	237 (188/49)	711 (564/147)	Non-cancerous controls (Age-,sex-matched; hospital-based)	57.9/58.4	Asian	Combined	Japan	39
Ni	2008	207 (189/18)	400 (362/38)	Healthy controls (Age-,sex-matched; population-based)	NA/NA	Asian	Larynx	China	30
Siraj	2008	223 (NA/NA)	513 (NANA)	Healthy controls (Age-matched; population-based)	NA/NA	Mixed	Thyroid	Saudi Arabia	36
Solomon	2008	126 (NA/NA)	100 (NA/NA)	Healthy individuals(population-based)	54.7/55.4	Asian	Oral cavity	India	37
Cao	2010	529 (339/190)	577 (367/210)	Healthy controls (Age-,sex-, ethnicity-matched; population-based)	46.1/45.4	Asian	Nasopharynx	China	22
Kruszyna	2010	131 (131/0)	250 (250/0)	Healthy controls (population-based)	59.4/56.8	Caucasian	Larynx	Poland	27
Rodrigues	2010	100 (84/16)	100 (76/24)	Non-cancerous controls (hospital-based)	59.5/43.6	Mixed	Combined	Brazil	34
Fard-Esfahani	2011	154 (34/120)	198 (50/148)	Non-cancerous controls (hospital-based)	NA/NA	Mixed	Thyroid	Iran	24
Prasad	2011	97 (27/70)	241 (116/125)	Healthy controls (population-based)	NA/NA	Asian	Thyroid	India	32
Sailasree	2011	130 (88/42)	139 (92/47)	Non-cancerous controls (Age-,gender-matched; hospital-based)	58.0/57.0	Asian	Oral cavity	India	35
Supic	2011	96 (72/24)	162 (130/32)	Non-cancerous controls(Age-, gender-, ethnicity-matched; population-based)	58.0/58.0	Caucasian	Oral cavity	Serbia	38
Tsai	2011	620 (583/37)	620 (572/48)	Healthy controls (Age-, gender-, habits-matched; population-based)	65.5/63.5	Asian	Oral cavity	China	40
Galbiatti	2012	322 (280/42)	531 (384/147)	Healthy controls (population-based)	NA/NA	Mixed	Combined	Brazil	25
Ozdemir	2012	60 (11/49)	50 (21/29)	Healthy controls (population-based)	55.3/68.6	Mixed	Thyroid	Turkey	31
Vylliotis	2013	160 (NA/NA)	168 (NA/NA)	Healthy controls (Age-, gender-, ethnicity-, working environment-matched; population-based)	58.5/54.7	Caucasian	Oral cavity	Greece	42
Kweon	2014	2194 (404/1790)	1669 (812/857)	Healthy controls (Age-, gender-, matched; population-based)	50.6/52.2	Asian	Thyroid	Korea	44

NA: not available

**Table 2:** Distribution of *MTHFR* C677T genotype among cancer cases and controls.

			**Cases**			**Controls**			**HWE (control)**	
**First Author**	**Year**	**Genotyping method**	**TT**	**CT**	**CC**	**TT**	**CT**	**CC**	**Chi-squre**	**P**
Weinstein	2002	PCR-RFLP	15	53	67	15	62	69	0.038	> 0.05
Kureshi	2004	PCR	0	12	22	4	18	32	0.420	> 0.05
Capaccio	2005	PCR	14	33	18	18	46	36	0.242	> 0.05
Neumann	2005	PCR	35	244	258	51	216	278	0.914	> 0.05
Vairaktaris	2006	PCR-RFLP	6	76	28	10	65	45	4.065	< 0.05
Hsiung	2007	PCR	149 (a)	-	128	306 (a)	-	218	-	-
Reljic	2007	PCR-RFLP	9	27	45	8	59	35	6.074	< 0.05
Suzuki	2007	Taqman	36	113	88	128	331	252	1.121	> 0.05
Ni	2008	PCR-RFLP	64	95	48	61	187	152	0.078	> 0.05
Siraj	2008	PCR	1	18	30	13	126	372	0.351	> 0.05
Solomon	2008	PCR-RFLP	23	55	48	10	42	48	0.033	> 0.05
Cao	2010	PCR-RFLP	32	169	310	30	188	334	0.275	> 0.05
Kruszyna	2010	PCR-RFLP	10	52	69	20	104	126	0.052	> 0.05
Rodrigues	2010	PCR-RFLP	13	43	44	14	40	46	1.182	> 0.05
Fard-Esfahani	2011	Multiplex PCR	14	71	69	8	108	82	14.224	< 0.05
Prasad	2011	PCR-RFLP	1	10	86	1	12	228	3.311	> 0.05
Sailasree	2011	PCR-RFLP	1	8	92	1	29	108	0.400	> 0.05
Supic	2011	PCR-RFLP	14	32	50	16	66	80	0.193	> 0.05
Tsai	2011	PCR-RFLP	43	186	391	62	236	322	3.606	> 0.05
Galbiatti	2012	PCR-RFLP	45	147	130	55	250	226	1.358	> 0.05
Ozdemir	2012	Real-time PCR	7	25	28	3	14	33	0.781	> 0.05
Vylliotis	2013	PCR-RFLP	6	76	28	10	65	45	4.065	< 0.05
Kweon	2014	PCR-RFLP	422	1050	722	291	851	527	2.748	> 0.05

(a): TT+CT

**Table 3:** Main results of the pooled data in the meta-analysis

		**TT vs CC**				**(TT+CT) vs CC**				**TT vs (CT+CC)**			
	**No. (cases/controls)**	**OR (95%CI)**	**P**	**P (Q-test)**	***I***^***2***^	**OR (95%CI)**	**P**	**P (Q-test)**	***I***^***2***^	**OR (95%CI)**	**P**	**P (Q-test)**	***I***^***2***^
Total	6354/7944	1.16 (0.93-1.45)	0.187	0.001	54.5%	1.05 (0.90-1.21)	0.540	0.000	68.7%	1.14 (0.93-1.38)	0.201	0.006	48.9%
Ethnicity													
Caucasian	1265/1545	0.95 (0.72-1.26)	0.742	0.860	0.0%	1.05 (0.81-1.38)	0.702	0.013	60.7%	0.92 (0.71-1.20)	0.533	0.511	0.0%
Mixed	996/1968	1.41 (0.99-1.99)	0.054	0.489	0.0%	1.06 (0.85-1.33)	0.597	0.143	37.5%	1.38 (0.99-1.92)	0.056	0.415	0.2%
Asian	4093/4431	1.24 (0.80-1.91)	0.331	0.000	80.3%	1.02 (0.78-1.34)	0.878	0.000	82.7%	1.21 (0.86-1.70)	0.279	0.001	73.1%
Source of control													
PB	5225/6252	1.24 (0.94-1.63)	0.121	0.001	59.0%	1.10 (0.92-1.32)	0.280	0.000	72.6%	1.21 (0.97-1.51)	0.086	0.019	46.4%
HB	1129/1692	0.88 (0.66-1.17)	0.377	0.392	2.5%	0.92 (0.71-1.19)	0.512	0.084	51.3%	0.91 (0.63-1.34)	0.646	0.181	36.1%
Sample size													
<500	1400/1881	1.28 (0.97-1.68)	0.083	0.794	0.0%	1.07 (0.83-1.38)	0.613	0.001	62.2%	1.24 (0.95-1.60)	0.111	0.637	0.0%
≧500	4954/6063	1.09 (0.76-1.55)	0.641	0.000	80.6%	1.03 (0.85-1.23)	0.785	0.000	77.1%	1.08 (0.80-1.45)	0.630	0.000	76.5%
Smoking status													
Never-smoking	606/919	0.79 (0.45-1.37)	0.394	0.635	0.0%	0.90 (0.71-1.14)	0.376	0.605	0.0%	0.81 (0.52-1.27)	0.362	0.967	0.0%
Ever-smoking	1223/1013	0.73 (0.49-1.10)	0.134	0.018	82.1%	1.24 (0.59-2.58)	0.573	0.000	94.2%	0.86 (0.62-1.21)	0.392	0.091	58.3%
Drinking status													
Light-drinking	201/474	0.78 (0.32-1.90)	0.588	0.455	0.0%	1.05 (0.75-1.46)	0.795	0.431	0.0%	0.99 (0.56-1.73)	0.961	0.717	0.0%
Heavy-drinking	409/519	3.11 (1.52-3.02)	0.002	0.832	0.0%	1.94 (0.88-4.27)	0.099	0.001	82.1%	1.62 (0.46-5.73)	0.457	0.001	86.0%
Tumor type													
Oral cavity	1298/1406	1.03 (0.66-1.61)	0.883	0.099	43.8%	0.99 (0.67-1.46)	0.950	0.000	78.8%	0.99 (0.67-1.45)	0.939	0.168	34.0%
Combined	1653/2667	0.97 (0.74-1.28)	0.830	0.293	17.9%	0.95 (0.79-1.15)	0.613	0.079	45.0%	0.97 (0.73-1.29)	0.827	0.191	31.0%
Larynx	338/650	1.82 (0.51-6.44)	0.353	0.007	86.1%	1.37 (0.63-3.00)	0.432	0.006	86.8%	1.64 (0.64-4.17)	0.302	0.033	77.9%
Thyroid	2554/2669	1.11 (0.92-1.32)	0.275	0.416	0.0%	1.28 (0.89-1.84)	0.185	0.020	65.8%	1.16 (0.99-1.37)	0.063	0.457	0.0%
Nasopharynx	511/552	1.15 (0.68-1.94)	0.601	—	—	0.99 (0.78-1.27)	0.958	—	—	1.16 (0.70-1.94)	0.566	—	—

PB: population-based; HB: hospital-based

Heavy-drinking: >46 g ethanol/week
